# High-Voltage Electrodes in Moist Air Accumulate Charge That is Retained after Removing the Electric Field

**DOI:** 10.1021/acs.langmuir.3c02390

**Published:** 2023-11-30

**Authors:** Esohe Fawole, William D. Ristenpart

**Affiliations:** Dept. of Chemical Engineering, University of California at Davis, Davis, California 95616, United States

## Abstract

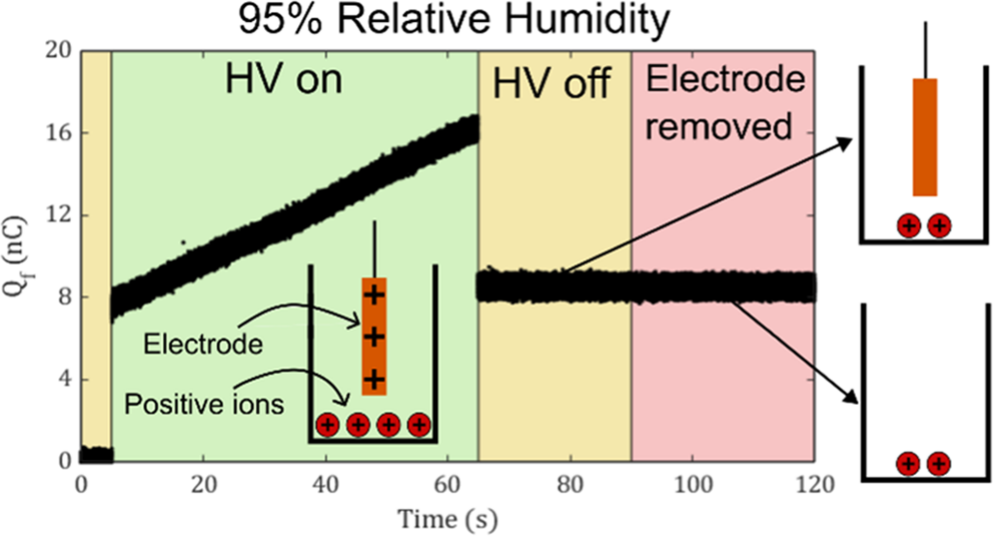

Applying a high voltage to a metal electrode that is disconnected from a circuit rapidly induces a capacitive charge, which quickly relaxes after removal of the applied voltage. Here, we report that if the electrode is placed in air at a sufficiently high relative humidity and provided the connection between the high-voltage supply and the electrode is composed of two different metals, the expected capacitive charge is followed by a gradual increase in charge. Surprisingly, this extra charge persists after the removal of the applied voltage and even after physically removing the electrode from the Faraday cup used to measure the charge. We report the median charge, average charge rate, and residual charge for different applied voltages, different metal–metal connections, and varied humidity. We interpret the results in terms of a proposed water ionization mechanism and discuss the implications of the findings for high-voltage fluidic systems.

## Introduction

High-voltage electric fields are used for a variety of systems and applications, including in lab-on-a-chip devices that require droplet merging,^1^ mixing,^2^ cell sorting,^3^ inkjet printing,^4^ and various biological applications.^5,6^ Many research groups^2,9−14^ have also examined the electrophoresis of charged droplets in a high-voltage, parallel-electrode system to probe the mechanism of droplet charge acquisition. A limiting prediction derived by Maxwell^15^ for the amount of charge *Q* a perfectly conducting sphere acquires upon contacting a planar electrode is
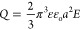
1where *a* is the radius of the sphere, *E* is the applied electric field, and εε_o_ is the permittivity of the surrounding fluid. Although experiments with charged droplet electrophoresis have generally corroborated these predictions, frequent deviations from theory have been encountered,^9−12,16,18,21−23^ even for solid conducting spheres.^17,19,20^

According to Maxwell’s theory, the magnitude of the charge acquired by the sphere should be independent of the polarity of the electrode, but numerous studies have indicated that aqueous droplets acquire more positive charge than negative charge for the same values of *a* and |*E⃗*|. For example, Eow et al.^9^ examined the phenomena of drop deformation and breakup under applied electric fields during the translation of a drop between two electrodes in insulating oil. For all applied electric fields tested, the velocity of the droplet after contacting the positive electrode was larger than that after contacting the negative electrode. Jung et al.^10^ studied the electrical charging of a water droplet at an electrode and observed a larger velocity of the droplet after contacting the positive electrode compared to the velocity from the negative electrode, indicating that the droplet regularly acquired more positive charge than negative charge. Im et al.^22^ examined the charging process of a bouncing droplet in silicone oil using a high-resolution electrometer and an image analysis method. They reported the negatively charged droplet velocity as 5.1 ± 0.08 cm/s (*n* = 77) and the positively charged droplet velocity as 5.9 ± 0.03 cm/s (*n* = 64). Elton et al.^11^ presented a current regression technique to measure the charge transferred to a droplet in silicone oil for a range of applied potentials and found that the positive charge was on average 69% greater than the negative charge. Finally, Elton et al.^21^ investigated the effect of droplet conductivity on the formation of bumps and craters on electrodes during charge transfer. They demonstrated that Joule heating due to high current densities during the charge transfer event locally melts the electrode, and the expansion of the plasma jet during dielectric breakdown pushes the molten material outward whereupon it cools and solidifies to form a crater. For the range of KCl concentrations tested, the ratio of positive charge acquired over negative charge acquired by a droplet was always greater than unity. The bump and crater model provided no explanation, however, for why the droplets received a more positive than negative charge.

In addition to this charging asymmetry, a pronounced time dependence of the droplet charge has also been observed.^18,23^ Elton et al.^18^ conducted a bounce-by-bounce analysis of droplet charge acquired after contacting an electrode, and they observed on average a 2.5% decrease in positive charge acquired and a 0.8% decrease in negative charge per 30 s of applied high voltage and concurrent droplet bouncing. No explanation is provided for this trend. Taken together, the aforementioned results indicate that there exist some unidentified confounding factors in the high-voltage systems that cause systematic deviations from the theoretical prediction of Maxwell’s charge.

One recurring theme is that none of the works listed above considered or reported the ambient air humidity. This omission is not surprising since extant theory for the charge considers only the electric properties of the droplet and insulating fluid in which it is immersed, not the surrounding air. There are important reasons to suspect that ambient humidity might play a role, however. At much larger scales, high-voltage transmission lines are used to transmit electrical energy from generators to substations, and numerous studies have examined the efficiency of transmission networks^7^ and methods to reduce “current leakage” of contaminated transmission lines under high humidity.^8^ More specifically for lab-on-a-chip systems, Yang et al.^23^ examined how induced surface charges on plastic or glass cuvettes varied with ambient humidity and thus affect the charge acquired by aqueous droplets immersed in silicone oil inside the cuvette. They also observed time-dependent changes in droplet charge acquisition from surface charges and reported a decrease in the absolute difference between negative and positive charges acquired at each electrode as the relative humidity increased. In trials over 50%RH, the effect of surface charges was minimized, and the charge disparity was significantly decreased. In their work, however, no mechanism is provided for the effect of humidity on surface charge development on the cuvette apparatus.

The goal of this work is to address the role of ambient humidity on charge acquisition in high-voltage systems. Toward this goal, we investigated a simplified system of just a single metal electrode suspended in air in an otherwise empty Faraday cup.

Surprisingly, applying a high-voltage potential to this seemingly simple system yielded anomalous charge accumulation dynamics, provided two criteria are satisfied: the ambient humidity is sufficiently high and a metal–metal junction between two metals is present. We show that although the applied voltage is constant, the measured voltage and thus charge in the Faraday cup increase with time, depending on the magnitude of the applied voltage and the relative humidity (RH). Furthermore, in trials where charge accumulation occurred, residual charge was left in the Faraday cup after shutting off the potential and even after removing the metal electrode. This observation suggests that the positive charge accumulated during the trial does not remain on the metal electrode but remains on the surface of the Faraday cup itself. We hypothesize that the results are consistent with a corona onset or “dark discharge”^24^ mechanism in humid air, and we discuss the implications for droplet charge acquisition experiments in high-voltage systems.

## Experimental Methods

**Figure 1:**
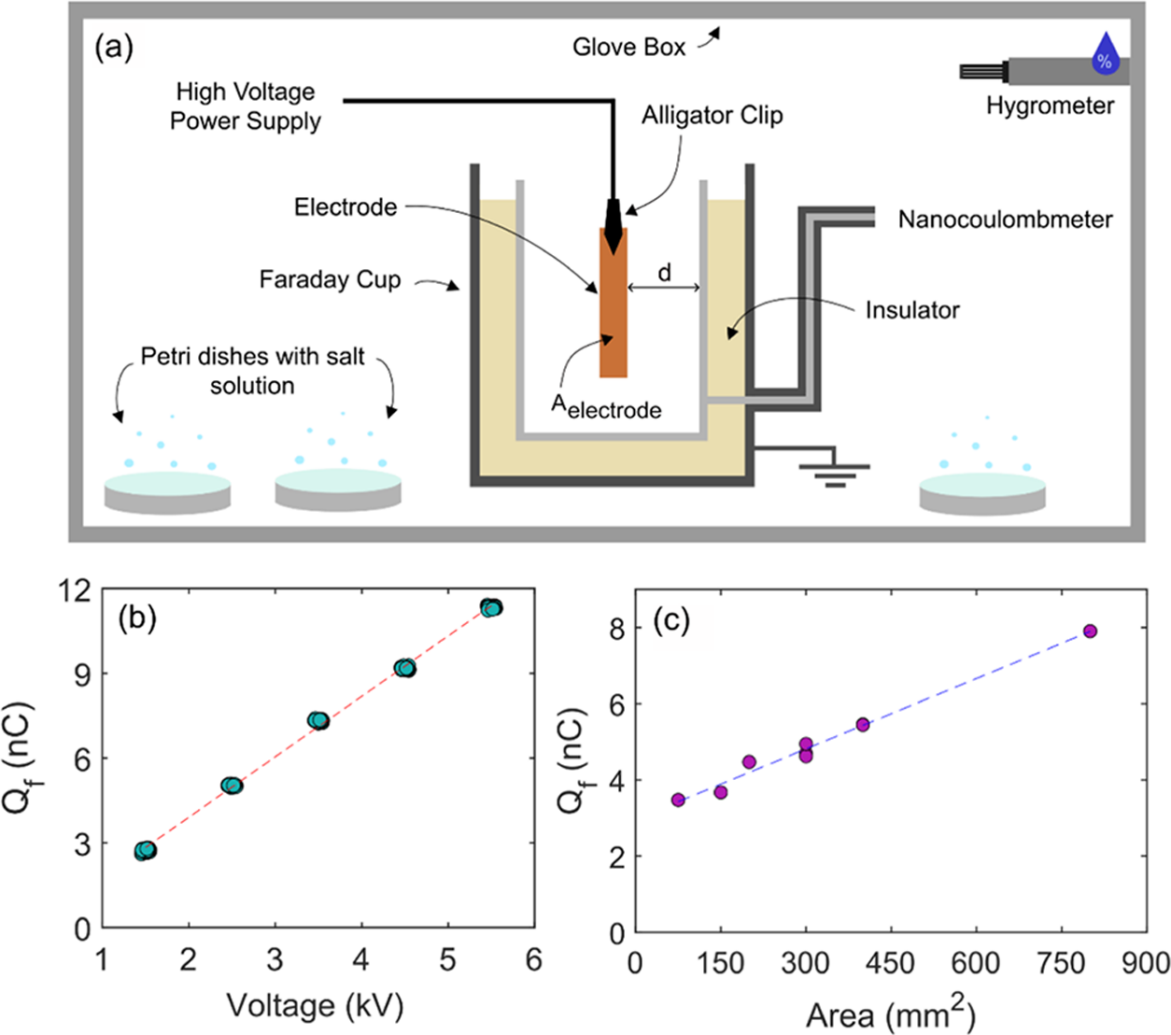
(a) Schematic of the experimental setup. The nanocoulombmeter reads the charge of the metal(s) in the Faraday Cup. (b) The steady induced charge of a stainless-steel alligator clip attached to a copper electrode (400 mm^2^) for different applied voltages at 50% relative humidity. There are 30 trials for each voltage (*N*_tot_ = 150). The distance *d* is approximately 3 cm for all trials. The red dashed line is the linear regression. (c) The steady induced charge of a stainless-steel alligator clip attached to different-sized copper electrodes with area *A*_electrode_ at 50% relative humidity and 3.3 kV applied potential. There are 3 trials for each tested area, so *N*_tot_ = 18. The blue dashed line is the linear regression.

The experimental setup for measuring charge under varied relative humidity conditions is illustrated in Figure 1a. A Faraday cup was placed inside a 0.29 m^3^ glovebox (approximately 0.9 m × 0.6 m × 0.5 m) with Petri dishes of saturated salt solutions to control the humidity. The Faraday cup (Advanced Energy Monroe, Model 284/22A) consisted of two concentric metal cups with a 1-in. insulating expanded polystyrene layer between them. The inner metal cup (inner diameter 6.5 cm, inner height 7 cm) is directly connected to a nanocoulombmeter (Advanced Energy, Model 284), whereas the outer metal cup is grounded.

When a charged object is placed inside the Faraday cup, current is generated by the electrons moving in response to the charged object. This current passes through the nanocoulombmeter, a charge amplifier that contains an operational amplifier (op-amp) integrator. In the op-amp integrating circuit, the output voltage is the integration of the input voltage over time. Placing a charged object in the Faraday cup generates the input voltage for the circuit, and integration is achieved by charging or discharging the capacitor in the feedback loop. Since *Q* = CV, the output voltage of the op-amp integrator is directly proportional to the charge of the object in the Faraday cup.

Preliminary validation experiments were conducted to test the impacts of the applied voltage and area of the metal electrode in relatively dry air. The classic expression for capacitive charge in a parallel-plate configuration is
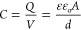
2where *C* is the capacitance, *Q* is the charge stored in a capacitor, *V* is the voltage, ε_o_ is the permittivity of free space, ε is the permittivity of air, *A* is the area of the electrode, and *d* is the distance between the plates.^24^ In our experimental apparatus, the relevant charge *Q* is the charge in the Faraday cup quantified by the nanocoulombmeter, denoted as *Q*_f_, while *A* is the area of the electrode and *d* is the distance between the electrode and the inner metal cup. Although our geometry is more complicated, we use eq 2 as an estimate to interpret the charge in the Faraday cup and test whether it scales linearly with the voltage and electrode area.

The analog output voltage signal from the nanocoulombmeter was recorded using a digital acquisition card at a rate of 1 kHz via LabVIEW software. Before starting a new experiment, the inner metal cup was wiped clean of debris with acetone, and the meter was tared using a momentary contact switch which discharges the integrator.

Metal sheets (copper, zinc, aluminum, nickel, titanium) were cut into 50 mm long, 8 mm wide, and 1 mm thick electrodes. For preliminary area validation tests, a 1 mm thick copper sheet was cut into separate rectangles, with dimensions 75 mm^2^ (10 mm × 7.5 mm), 150 mm^2^ (10 mm × 15 mm), 200 mm^2^ (20 mm × 10 mm), 300 mm^2^ (10 mm × 30 mm), 400 mm^2^ (20 mm × 20 mm), and 800 mm^2^ (20 mm × 40 mm). Prior to each experiment, the metal electrode and alligator clip were sonicated in isopropanol, acetone, and then water individually for 10 min each before being dried with pure nitrogen gas.

Saturated salt solutions of KNO_3_, NH_4_Cl, NaCl, K_2_CO_3_, and KC_2_H_3_O_2_ were prepared in 1 L of DI water (18.2 MΩ/cm) and were distributed roughly equally between Petri dishes. To maintain 30, 50, 70, 80, 90, or 95% RH in the glovebox,^25,26^ the Petri dishes of solution were left in the glovebox overnight, approximately 12 h. Experiments were initiated after the hygrometer (Fisher Scientific) maintained the desired relative humidity for an hour.

Before conducting a trial, a metal alligator clip was connected to the high-voltage power supply (Trek 610E) by an insulated copper wire, and an electrode was held in place at the mouth of the alligator clip. The metal alligator clip and electrode were suspended in the Faraday cup by an insulated copper wire, hanging approximately 1 cm above the inner cup surface. For each experiment, the meter was tared before background data was collected. After 5 s, high voltage was applied to the metal clip-electrode pair for either 1 min for copper electrodes or 5 min for aluminum, titanium, nickel, and zinc electrodes. Once the time limit was reached, the high voltage was shut off, and the alligator clip and electrode were removed from the Faraday cup. Data collection continued for 5–60 more s before concluding the trial. All metal–metal combinations tested in this study are shown in Table 1.

**Table 1:** Relative Humidity, Applied Voltage, and Number of Experimental Trials for All Tested Metal–Metal Combinations^a^

inspected metal combination	shape	tested voltage(s) (kV)	tested %RH	# of experiments
stainless-steel–copper	clip-electrode	1.5–5.5	50–100	603
	clip-tape	3.3	30–100	16
	clip-clip	3.3	100	6
stainless-steel–stainless-steel	clip-plate	3.3	100	16
	clip-tape	3.3	100	44
	clip-electrode	3.3	100	14
copper–aluminum	clip-tape	3.3	100	9
	clip-foil	3.3	100	3
	clip-electrode	3.3	100	9
copper–nickel	clip-electrode	3.3	100	9
copper–titanium	clip-electrode	3.3	100	7
copper–zinc	clip-electrode	3.3	100	5
copper–lead	clip-electrode	3.3	100	5
copper–tin	clip-electrode	3.3	100	14
copper–copper	clip-electrode	3.3	50, 100	21
	clip-wire	3.3	100	6
	clip-tape	3.3	50, 100	48
	clip-clip	3.3	100	10
copper–stainless-steel	clip-plate	3.3	100	5

a All experiments and trials were conducted at room temperature (23 °C).

## Results

Our preliminary validation experiments corroborated the validity of eq 2, at least for sufficiently low voltages and relative humidities. With a 1 kV step increase in applied potential, a linear relationship was observed between the applied potential and the measured charge in the Faraday cup (Figure 1b). Tests with increasing electrode area at a fixed potential also corroborated eq 2 (Figure 1c). The alligator by itself capacitively charged to about 3 nC (the “zero area” intercept in Figure 1c), and adding flat copper electrodes of increasing area yielded a linear increase in measured charge. The larger amount of apparent noise in the area plot was presumably due to small sizing variations in the metal electrodes. It is important to note that all trials in Figure 1b,c were conducted at 50% relative humidity; at higher RH, as shown below, the assumption of a single charge value after application of the high voltage no longer holds.

**Figure 2:**
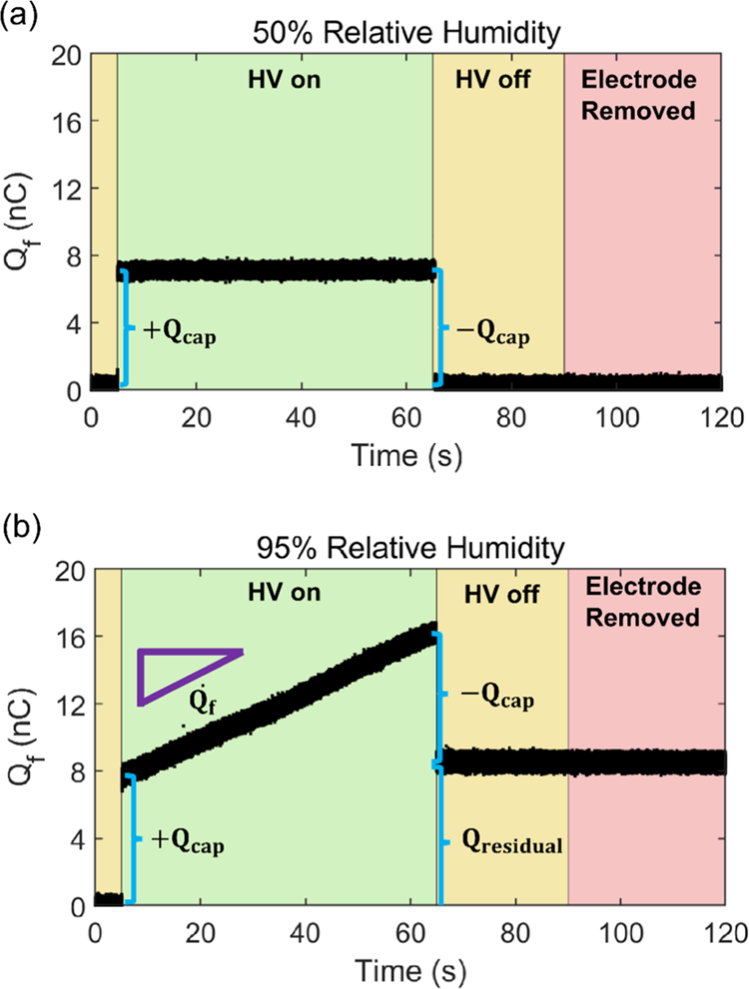
(a) Representative example of charge acquired by a stainless-steel alligator clip and copper electrode in the Faraday cup at 50% relative humidity. Yellow regions denote time periods where the high voltage is deactivated; the green region denotes the time period when the high voltage is applied; the red region denotes the time period after the electrode and clip were physically removed from the cup. (b) Representative example of charge acquired by the same clip-electrode pair in the Faraday cup at 95% relative humidity. Colors same as in (a).

A representative trial of the transient dynamics of charge acquired by a stainless-steel alligator clip and copper electrode at 50% and 95% RH reveals a significant impact of humidity (Figure 2). With the relative humidity maintained at 50%, application of a 3.5 kV potential to the clip and electrode after 5 s immediately induced a positive capacitive charge (+*Q*_cap_), in this case approximately 7 nC (Figure 2b). For the duration of the applied voltage, the charge in the Faraday cup remained constant at 7 nC. After 60 s, the applied voltage was removed and the charge in the Faraday cup immediately dropped by the equal and opposite decapacitive charge (−*Q*_cap_), as expected for a capacitive charge in a system connected to the ground. After approximately 90 s from the beginning of the trial, the clip and electrode are removed from the Faraday Cup, with no apparent impact on the near-zero charge, as expected.

Qualitatively different results were obtained at 95% RH using the same electrode system (Figure 2b). After the potential was applied, the charge in the Faraday cup immediately jumped to a similar capacitive charge near 7 nC. In contrast to the 50% RH trial, however, the measured charge steadily increased over the duration of the applied voltage, increasing to about 15 nC over 60 s, i.e., more than doubling. No visual or auditory evidence of dielectric breakdown was observed during the high-voltage application. Upon removal of the high voltage, the charge in the cup immediately dropped by an amount close to the initial capacitive charge of 7 nC, but leaving a significant residual charge of 15 nC, denoted here as *Q*_residual_. A further 30 s after removal of the high-voltage field, the stainless-steel clip and copper electrode were both physically removed from the Faraday cup; surprisingly, the residual charge in the cup remained unaffected by the removal. We emphasize that this behavior is very different from what occurs when a charged object (e.g., a piece of plastic with static charge) is removed from the Faraday cup since removal of the charged object causes the measured charge in the cup to return to zero. The implication of the data in Figure 2b is that the charge associated with *Q*_residual_ was not on the metal clip nor electrode but was instead on the surface of the Faraday cup itself. At no point during the 95% relative humidity trial did the charge in the Faraday cup return to zero during our measurements. This observation suggests that the charge in the cup would remain indefinitely provided no adjustments are made to the system.

**Figure 3:**
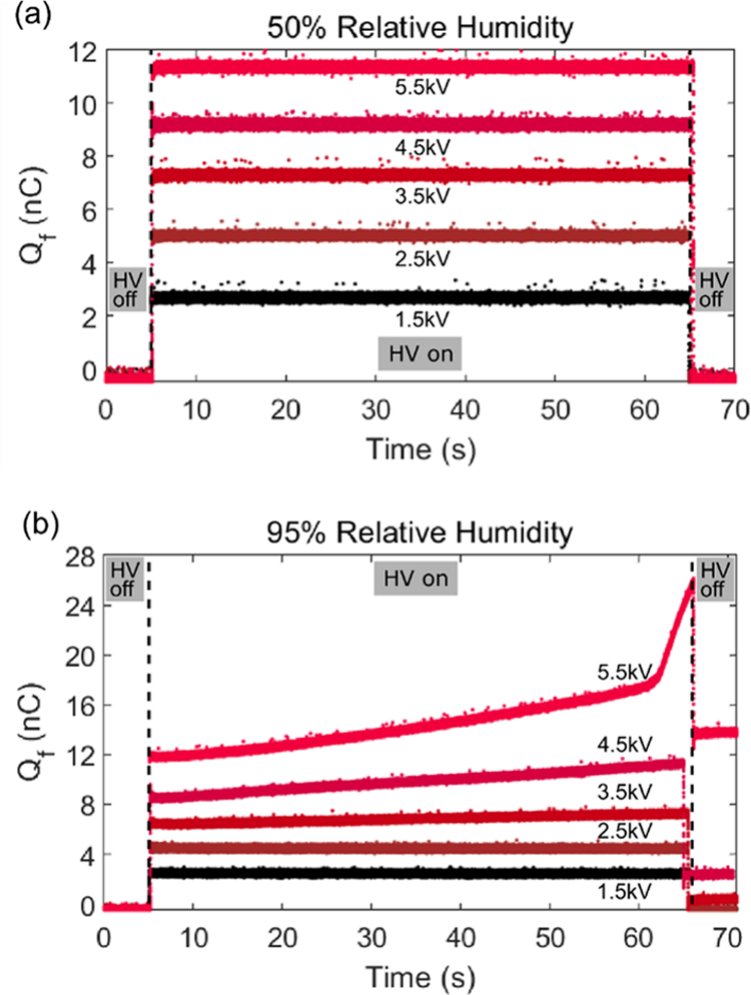
Representative trials of charge acquired by a stainless-steel alligator clip and copper electrode in the Faraday cup at varied voltages for (a) 50% relative humidity and (b) 95% relative humidity. Vertical dashed lines indicate when the high voltage was applied and deactivated.

Further experiments confirmed that the behavior illustrated in Figure 2 is qualitatively reproducible under a wide range of conditions. Representative trials of charge acquired by a stainless-steel alligator clip and a copper electrode at different applied potentials are shown in Figure 3. At 50% relative humidity, voltages ranging from 1.5 to 5.5 kV were applied to the clip and electrode for 60 s. For the duration of each applied voltage, the charge in the Faraday Cup remained constant, with the magnitude of the induced charge proportional to the applied voltage, as expected via eq 2 (Figure 3a). In each case, after the applied voltage was removed, the charge measured in the Faraday cup immediately decreased back to zero. In contrast, the trials at 90% relative humidity revealed a voltage dependence (Figure 3b). Here, the charge remained constant over the 60 s that 1.5 and 2.5 kV were applied. However, in the trial at 3.5 kV, the charge slowly increased with time. A faster increase was observed for 4.5 kV. For the 5.5 kV trial, the charge accumulation was most rapid, and then, approximately 62 s after application of the high voltage, the rate of charge accumulation suddenly and drastically increased before the applied potential was deactivated. We emphasize that no intentional alterations to the Faraday cup or the electrode occurred while the potential was applied. Similar to the result highlighted in Figure 2b, here for the trials with a positive charge rate (3.5, 4.5, 5.5 kV), the residual charge was clearly nonzero, with the magnitude of the residual charge proportional to the applied potential.

**Figure 4:**
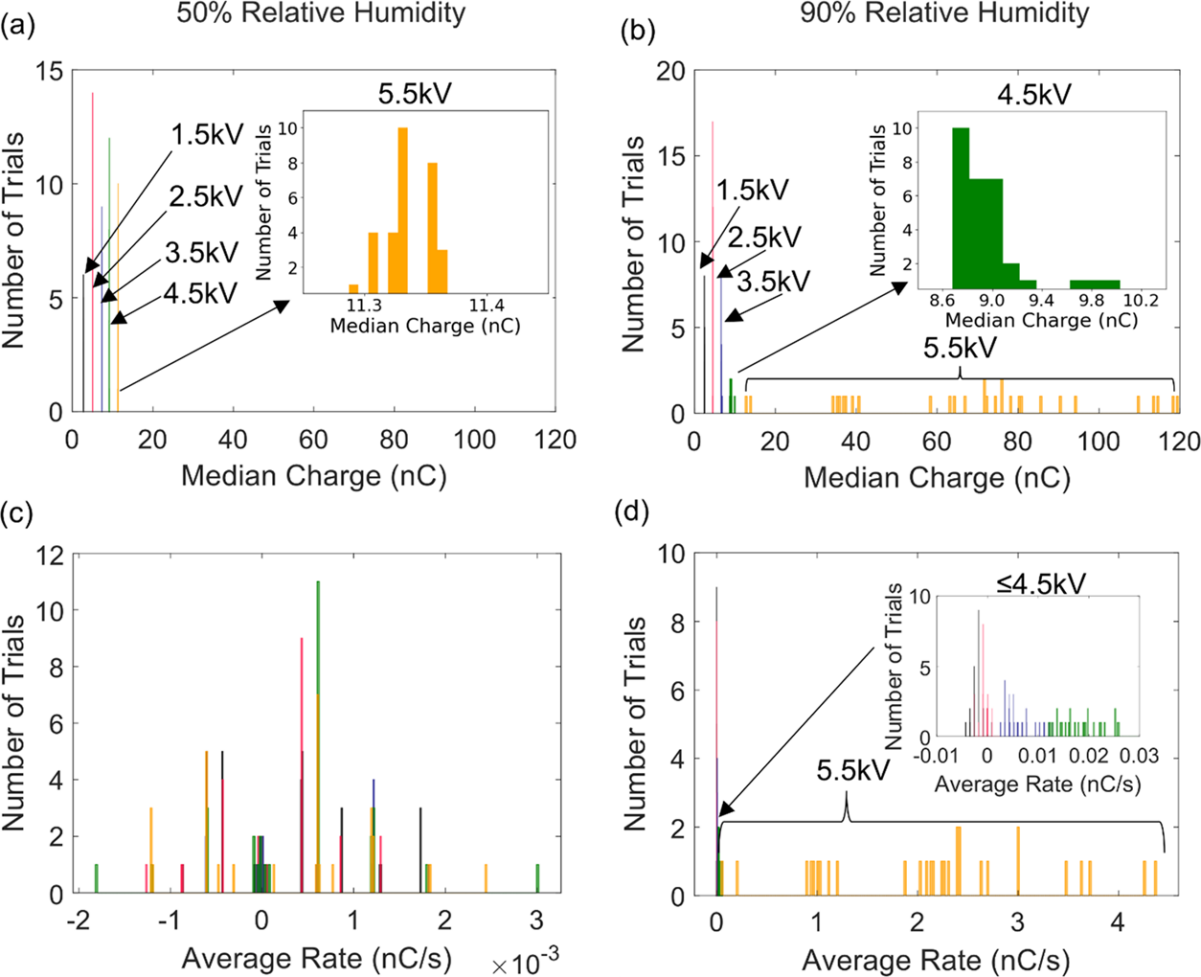
(a, b) Histograms of the median induced charge for a stainless-steel alligator clip and copper electrode for (a) 50% relative humidity and (b) 90% relative humidity. (c, d) Histograms of the average charge accumulation rate for a stainless-steel alligator clip and copper electrode for (c) 50% relative humidity and (d) 90% relative humidity. In each, the colors denote different applied voltages: black, 1.5 kV; red, 2.5 kV; blue, 3.5 kV; green, 4.5 kV; and orange, 5.5 kV.

To probe the mechanism of charge accumulation and residual charge, we performed a systematic series of replicate experiments to test the quantitative reproducibility of the charge dynamics observed in Figure 3, again using a stainless-steel clip and copper electrode. For each of the 5 voltages (1.5 2.5, 3.5, 4.5, and 5.5 kV) tested, 30 trials each were conducted at 50, 70, 80, and 90% RH. The median charge and average rate at 50 and 90% RH are plotted in Figure 4. The median charge was calculated by considering only the charge measurements during the 60 s application of the high voltage. The average charge rate was calculated by using a linear regression to the charge data over the 60 s the potential was applied; note this procedure yields only an estimate of the average charge rate for the highest voltages in trials that exhibited large variations in the slope (as illustrated in the 5.5 kV curve in Figure 3b).

At 50% relative humidity, the median charges for all five voltages tested were highly consistent across 30 trial replicates, with standard deviations on the order of 10^–2^ nC (Figure 4a). At 90% RH, however, the distribution of the median charge is wider (Figure 4b). For the 1.5 and 2.5 kV trials, the distribution around the median charge at 90% RH had comparable standard deviations as 50% RH. For 3.5 and 4.5 kV trials, the median charge distribution is slightly wider, with an order of magnitude increase in standard deviation between the two voltages at 90% RH. Trials at 5.5 kV had the largest median charge distribution range, from 13 to 119 nC; 20 out of 30 trials had a distinctly different median charge value, showing the irreproducibility at high (90%) relative humidity. Similar RH-dependent behavior was observed with the rate of charge (Figure 4c,d). At 50% RH, the average charge rates for all voltages tested were nominally zero, on the order of 1 × 10^–3^ nC/s, resembling a normal distribution (Figure 4c). At 90% RH (Figure 4d), trials at ≤4.5 kV appear as one lumped distribution around zero nC/s. Upon closer inspection, the average charge rate for these trials falls between −0.015 and 0.027 nC/s, an order of magnitude larger than the 50% RH rates, with standard deviations between 0.001 and 0.011 nC/s. At 5.5 kV, trials exhibit a wide distribution ranging from 1 to 4.5 nC/s. The irreproducibility of the rate of charge accumulation for 5.5 kV is evident by the low count numbers over the range of rate values and large standard deviation (1.11 nC/s), consistent with the irreproducibility of median charge at 5.5 kV. Overall, trials at 90% RH had greater median charges and average charge rates despite the increase in deviation at higher voltages.

**Figure 5:**
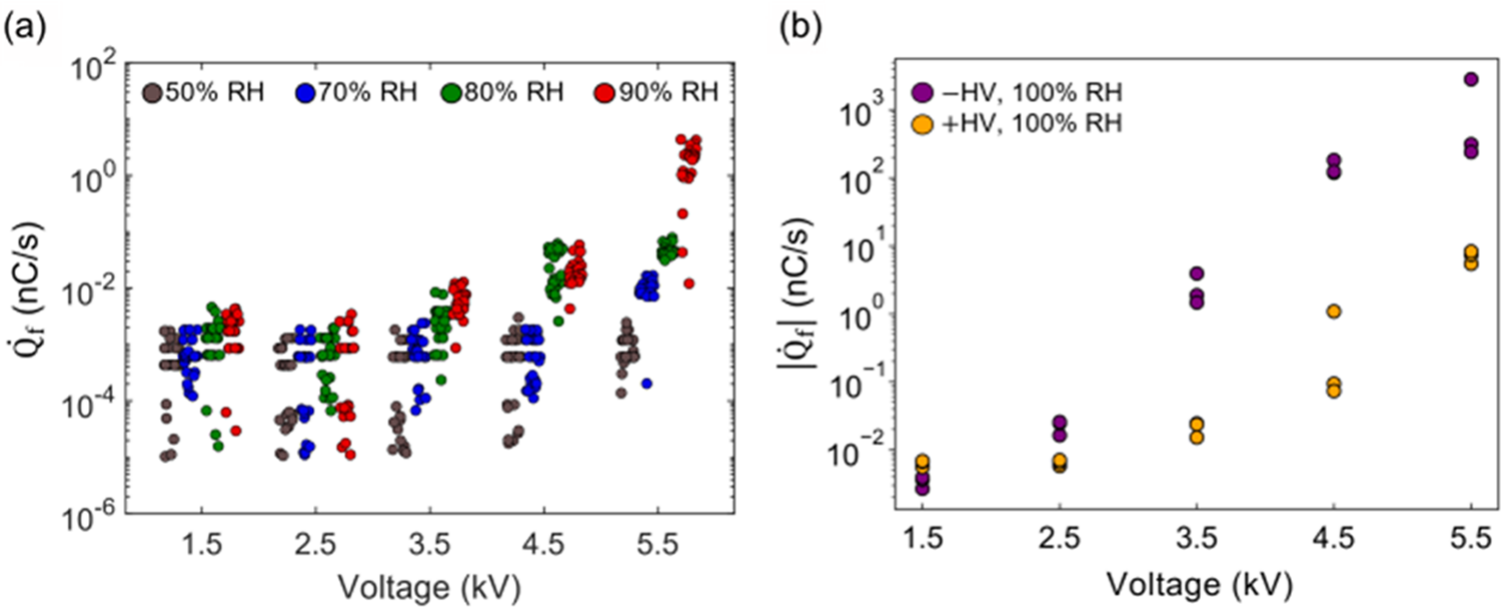
(a) Initial charge accumulation rate for different applied voltages at different relative humidity. All trials were conducted with a stainless-steel alligator clip and a copper electrode. For each relative humidity at a specific applied voltage, 30 trials were conducted (*N*_tot_ = 120). At each applied potential, data points are offset horizontally for clarity. (b) Absolute charge rate for different positive (orange) and negative (purple) applied voltages at 100% relative humidity. There are 3 trials for each positive and negative applied voltage (*N*_tot_ = 30).

The charge data in Figure 4 highlight the results at 50 and 90% RH. A summary of the initial charge rate for all four tested RH values, comprising 600 trials in total, is presented in Figure 5a. Here, we focus on the initial rate of charge accumulation (immediately after *t* = 0), omitting the sudden accelerations in the charge rate typically observed at high voltages and high RH. For the 1.5 and 2.5 kV trials, the charge rate increased with increasing relative humidity, although the rate for each humidity tested mostly remained on the order of 10^–3^ nC/s. At higher voltages, the increase in rate with increasing humidity was larger for each 1 kV step.

The initial charge rate for negative applied potentials at 100% RH was also examined (Figure 5b). Similar to the results of positive applied potentials, an increase in charge rate as the applied negative voltage is increased was observed. Below 3.5 kV, differences in rate between negative HV and positive HV were less than an order of magnitude; however, negative charge rates were approximately 2 orders of magnitude larger than positive charge rates above 3.5 kV. This result suggests a strong polarity dependence in the mechanism driving the high-humidity charge accumulation.

**Figure 6:**
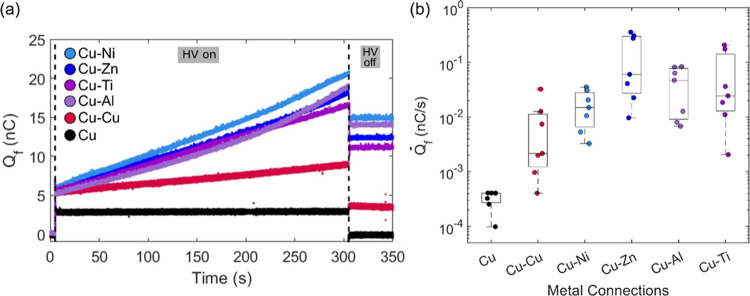
(a) Representative examples of charge accumulation for an isolated copper alligator clip (black points) or for different copper alligator clip-metal electrode pairs (respective colored points). Vertical dashed lines indicate when the potential was applied and removed. (b) Boxplots of the rate of charge accumulation for the types of metal connections illustrated in (a). There are 7 trials in each column. For clarity, each data point is randomly offset horizontally from the center of the column. All trials were at 3.3 kV applied potential and 100% relative humidity.

The above experimental results all involved a stainless-steel alligator clip and a copper electrode. We initially hypothesized that similar results would be obtained for any type of conductive metallic electrode, but our experimental tests with different types of metals reveal a pronounced sensitivity to the type of metal. Specifically, we tested an isolated copper alligator clip not attached to any electrode as well as a copper clip connected to a copper, aluminum, nickel, titanium, or zinc electrode (Figure 6). At 50% RH, the stand-alone copper clip and all copper clip/metal electrode pairs exhibited nominally zero charge rates, on the order of 10^–3^ nC/s (data not shown). Similar results were observed for the stand-alone copper clip at 95% RH (Figure 6a, black). Presumably due to the smaller surface area, the median charge of the clip by itself was approximately 66% lower than the median charge of the clip-electrode pairs. In contrast, at 95% RH, the five tested metal electrodes all had positive charge rates over the 5 min the potential was applied and then nonzero residual charge once the potential was removed (Figure 6a). We hypothesize that the copper clip–copper electrode pair (Figure 6a, red) exhibited a lower charge rate than the other metal pairs due to similar metal composition between the clip and electrode, although we cannot rule out the possibility of minor compositional differences. The results of the dissimilar metal clip-electrode pairs indicate that charge accumulation does not only occur between the SS clip and copper electrode.

**Figure 7:**
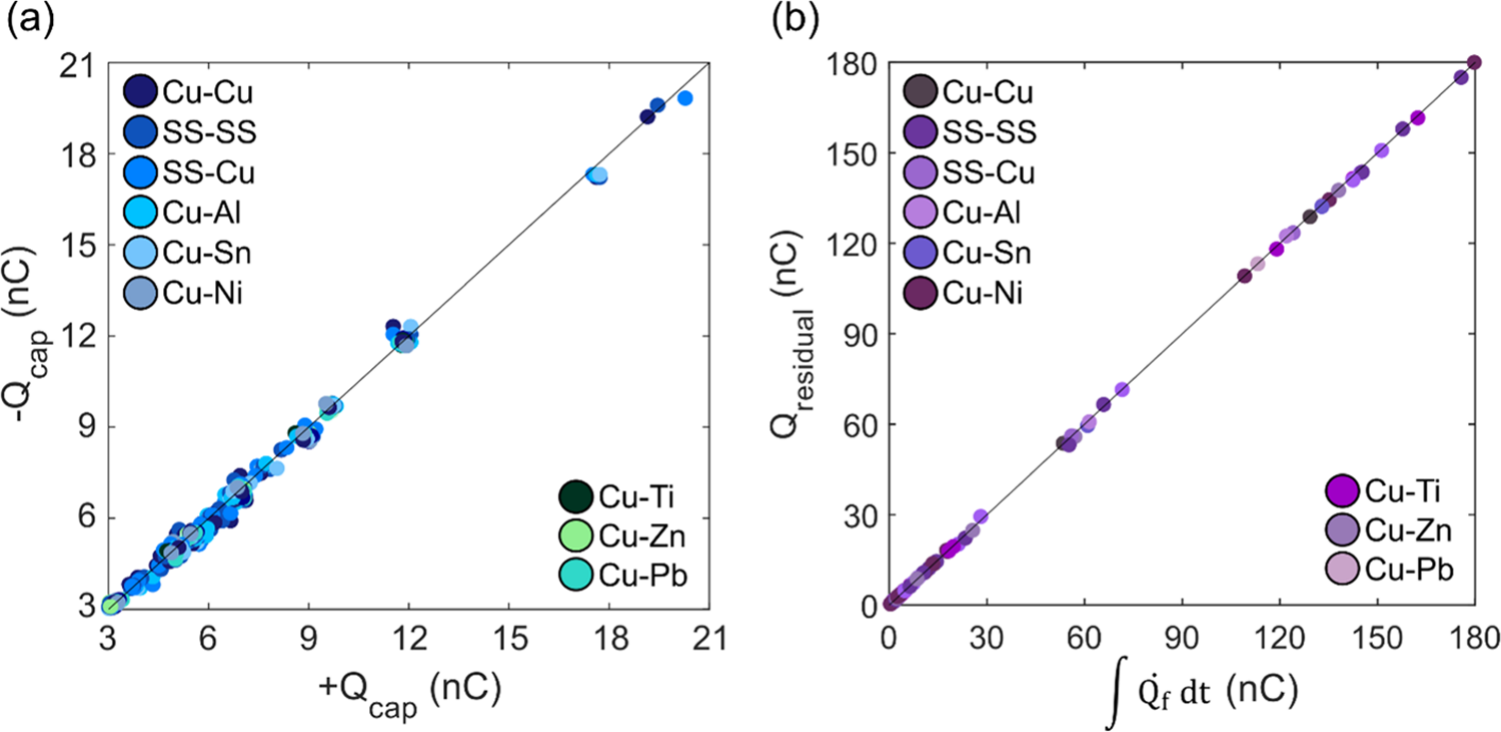
(a) Correlation between the induced capacitive charge (+*Q*_cap_) and subsequent decapacitive charge (−*Q*_cap_) for *N* = 850 trials. Shades of blue represent different types of metal clip–metal electrode pairs. The solid line indicates slope of unity. (b) Correlation between the residual charge (*Q*_residual_) and the integral of the charge accumulation rate (*Q̇*_f_) over the duration of applied potential for *N* = 250 trials. Shades of purple represent different types of metal clip–metal electrode pairs. The solid line indicates the slope of unity.

Figure 6b shows the equivalent charge rates for all isolated copper clip and clip-electrode pair trials at 95% RH. The isolated copper clip trials had nominally zero charge rates. Regardless of the humidity conditions, no charge rate larger than 5 × 10^–4^ nC/s was ever observed for the isolated clip. For similar and dissimilar metal connections at 100% RH, charge accumulation was detected. While the copper clip-copper electrode junction exhibited some positive charge rates, the rate was often less than 6 × 10^–3^ nC/s. Compared to an isolated clip or similar metal connection, dissimilar metal connections were observed to have consistent positive charge rates as well as the largest magnitude of charge accumulation. One recurring feature exhibited in all experiments was that the residual charge appeared to have a similar magnitude to the charge accumulated over the duration of the applied high voltage, independent of the capacitive charge acquired immediately after the application of the high voltage. To assess this relationship quantitatively, we first analyzed the relationship between the capacitive charge and the decapacitive charge, identified in Figure 2, for *N* = 850 trials (Figure 7a), consisting of a range of metal–metal combinations shown in Table 1. The capacitive and decapacitive charges all mostly lie on the line with a slope of unity, indicating that the capacitive charge experienced when the voltage is applied is equal to the decapacitive charge after deactivation, regardless of metal composition, size, or shape, relative humidity, duration of trial, rate of charge accumulation, or residual charge. The relationship between the residual charge left in the cup and the integral of the charge rate over the time the high voltage is applied is shown in Figure 7b. All *N* = 250 trials in this figure are trials in which charge accumulation occurred, i.e., where *Q̇*_f_ was greater than 1 × 10^–3^ nC/s. The trials here were at 50–100% relative humidity and are composed of all metal–metal combinations. Again, the data are well fit by the line with a slope of unity, indicating that the value of the residual charge is equivalent to the integral of the charge rate while the potential was applied. In other words, the charge accumulated over the length of time the potential is applied, in excess of the initial capacitive charge, is in all cases equal to the residual charge left in the Faraday cup.

**Figure 8:**
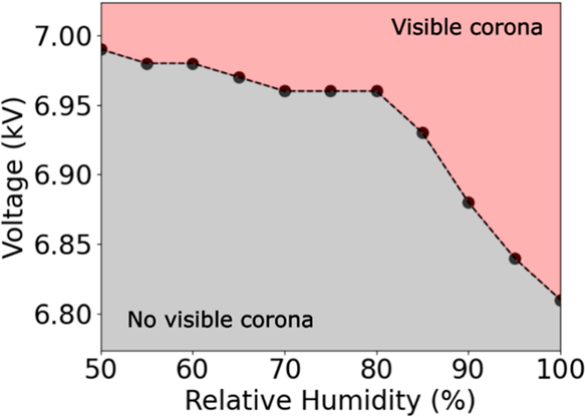
Visible corona threshold for a copper electrode and stainless-steel clip in the Faraday cup versus the relative humidity. Here, “visible” denotes visible by the naked eye, which invariably occurred concurrently with audible breakdown noises.

We emphasize that in all of the previous experiments we never observed any visible or audible corona discharge. A corona is a weakly luminous, partially ionized gas discharge, which usually appears at atmospheric pressure near sharp points, edges, or thin wires of one electrode where the electric field is sufficiently large.^24,27,28^ In our setup, the sharp corners of the rectangular copper electrode potentially generate the nonuniform electric fields necessary to initiate corona. To probe in our system what voltages are necessary to induce a visible corona in our apparatus, we systematically increased the applied voltage while holding relative humidity constant, until visual and auditory effects were apparent (Figure 8). The visual corona threshold remained relatively constant at approximately 6.95 to 7.0 kV for lower humidities. Above 80% RH, we observed a gradual decrease in threshold voltage, albeit dropping only 0.2 kV from 50% RH to 100% RH. Importantly, all trials reported in Figures 1–6 were conducted at potentials well below the visible corona threshold.

## Discussion

From the data collected, it is evident that high humidity, sufficient applied potential, and a dissimilar metal connection are needed to observe charge accumulation. The obvious question is why? What is the mechanism of charge accumulation?

There are a few potential mechanisms that do not seem to align with our observations. Ducati et al.^29^ offer an ion partitioning mechanism for their charge accumulation observations on a metal electrode. Their physical setup includes isolated, cylindrical metal samples placed within an outer copper-plated-brass cylinder separated by polyethylene foam rings. An aluminum box fitted for gas circulation is used to alter the relative humidity, and no voltage was applied to either metal throughout their study. They describe the charge buildup on the isolated metal as water molecules contributing OH– or H+ ions to the oxide-coated metal surface. Depending on the oxide layer’s nature and state, metal charging under high humidity is the outcome of surface reactions where adsorption and desorption of water carry charge to and from the metal surface, imparting excess charge to the isolated metal. Ducati et al. also examined charging on an SS-dielectric-aluminum dissimilar metal capacitor where the dielectric between the metals had a high capacity of water vapor absorption. According to the ion partitioning mechanism, the ions segregate onto the two pieces of metal, so the metal system itself remains electrically neutral. In our experiments, however, a potential is applied to the isolated electrode, and the charge apparently accumulates on the Faraday cup itself (as evidenced by the retention of charge in the Faraday cup even after the electrode is removed). Similarly, Lax et al.^30^ also observed the accumulation of voltage on isolated metal cylinders during high relative humidity exposure, both in controlled lab conditions and ambient outdoor conditions. Their physical setup was similar to that used by Ducati et al., with no voltage applied to any metals in their work; however, they tested and compared several metals and metal composites. Lax et al. reported voltage accumulation between two dissimilar metals in cases where RH > 60% and observed 0.65 V accumulate on the zinc cylinder after approximately 1600 s, a significantly longer time scale compared to our work. No charge measurements were reported, and a mechanism for voltage accumulation was not provided. Given these ambiguities and experimental differences, it is difficult to rationalize our results in terms of ion partitioning near the metal–metal interface.

Another potential mechanism involves the dielectric breakdown of the humid air between the copper electrode and the steel Faraday cup. Here, we assess two common types of dielectric breakdown mechanisms: Townsend^31^ (occurs in uniform electric fields) and corona (occurs in nonuniform electric fields). Both mechanisms are initiated with an electron avalanche, where electrons are initially generated either from ultraviolet (UV) irradiation of the cathode^27^ (Townsend) or from a small volume of space at the anode that produces a high enough field strength to cause ionization by collision (corona).^24^ In an electric field, these electrons are accelerated toward the anode and collide with molecules, generating successive avalanches where the head is made of electrons and the long tail is populated by positive ions. The space charge of slow-moving positive ions enhances the electric field between the electrodes and results in rapid current growth, leading to breakdown.

The experimentally determined relation between the breakdown electric field strength and the pressure spacing product (pd) for Townsend discharge is usually referred to as the Paschen curve.^27,32^ Given our gap distance (3 cm) and pressure spacing product (30.4 bar mm), the breakdown voltage for our interelectrode air gap determined by Paschen’s curve would be 100 kV, 2 orders of magnitude greater than our tested voltages (1.5–5.5 kV). Considering humidity, studies have shown that the breakdown voltage of air increases with increasing relative humidity,^33−38^ suggesting that if currents were responsible, we would see less charge accumulation at higher humidities—the opposite of our observations. In combination with the observed lack of any audible noise or light in all trials, plus our experimental corroboration that corona discharges did occur at much higher applied potentials, we conclude that our experiments occurred at field strengths below the breakdown regime for the uniform electric field gap space.

Although no visible corona were observed, another possibility is that a “pre-corona” current or “pre-breakdown regime current” was instead responsible for the observed charged accumulation. The average current growth to breakdown (pre-breakdown regime) as a function of the applied voltage for uniform electric fields was qualitatively described by Townsend. Initially, there is a proportional increase in the current as the applied voltage is increased, which qualitatively matches our results in Figure 5. In regard to the effect of humidity, a reduction of electrons’ kinetic energy due to frequent collision with H_2_O has been observed in humid air.^33^ Consequently, higher electric fields, keeping interelectrode distance and electrode geometries constant, are required to initiate electron avalanches and subsequent current growth during pre-breakdown. Our results indicate the opposite; holding the applied voltage constant, an increase in current was observed as the relative humidity increased. Therefore, it is difficult to interpret observed charge accumulation in terms of the pre-breakdown regime of the Townsend mechanism.

DC corona discharge behavior is likewise affected by changes in relative humidity. As the relative humidity in the air gap between electrodes is increased, three key trends are observed: (1) the corona onset voltage decreased,^39,42,45−49^ (2) the steady corona current increased for low DC voltages (<7 kV for *d* = 1 cm)^39,42,45,46^ and decreased for high DC voltages,^40−45,50^ and (3) the positive ions’ mobility decreased.^42−46,49^ In our work, the corona onset current (charge rate in Figure 5a) at 5.5 kV, 70%RH was comparable to the onset current at 3.5 kV, 90% RH indicating an increase in relative humidity decreased the voltage required to initiate the observed current, corroborating the first key trend described above. For each voltage tested, the corona onset current increased with increasing relative humidity as observed by previously mentioned studies.^39,42,45,46^

Under DC voltages, ionization products have sufficient time to wander in the gap and accumulate in space.^38^ This ion drift contributes to the continuous unipolar current in the initial stage of corona discharge.^51^ While electrons are responsible for the total current at the anode surface, positive ions carry the total discharge current away from the anode since negative ions have lower mobility.^52^ A steady positive current under DC voltages is similarly observed in our work. As the applied voltage increased from 1.5 to 5.5 kV for a 1 cm gap space, an increase in the onset current was measured. This relationship, discussed in previous studies,^39−46^ is due to the increase in the electric field at the surface of the anode which leads to an increase in the total positive space charge, more ionizing collisions, and a greater number of charged ions contributing to the onset current.^47^

Analogous to positive corona, an increase in current was observed as the applied negative voltage increased; however, orders of magnitude differences in current between negative HV and positive HV were prominent in magnitudes above 3.5 kV (Figure 5b). This difference can be explained by electrons, in addition to negative ions, contributing to the negative corona current^42^ and lower susceptibility of negative ions to hydration compared to positive ions at high relative humidity.^53^ Importantly, for both negative and positive corona, the effect of ion mobility on corona current at high humidity was found to be negligible for low applied voltages.^42,54^ Rather, the ease with which ions are generated has a considerable impact on the humid corona current. Mass spectrometry of ions extracted from corona discharges at high humidity^55,56^ indicate that the dominant positive ions are [H_3_O]^+^·[H_2_O]_*n*_, which are formed from water clusters, [H_2_O]_*n*_ with 2 ≤ *n* ≤ 6. Compared to other common air molecules, water clusters have been found to have lower ionization potentials.^55,56^

**Figure 9:**
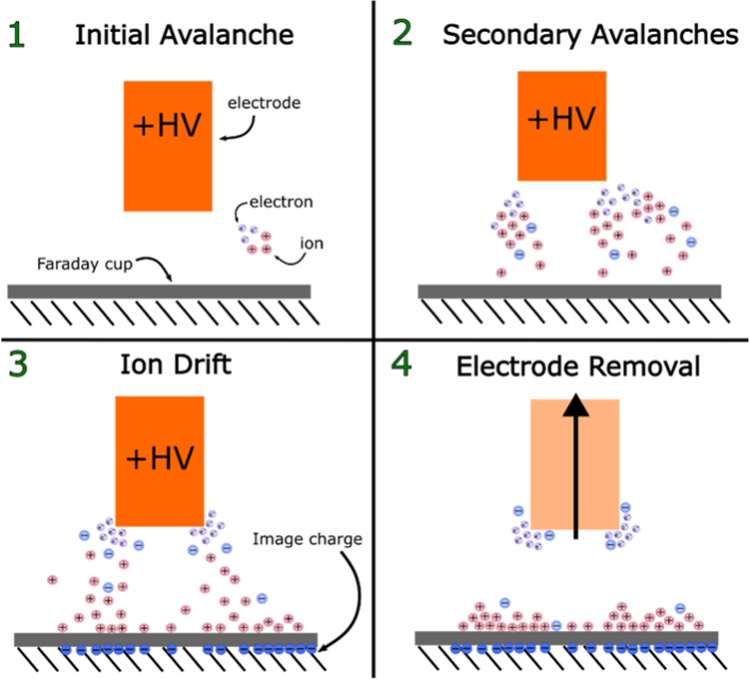
Schematic of the proposed corona onset mechanism for charge accumulation on the HV electrode in the Faraday cup.

To summarize, we interpret the observed charge accumulation in the Faraday cup in terms of the following mechanism (cf., Figure 9). (1) A small volume of space at the corners of the copper electrode produces the necessary field strength for ionization by collision, producing a free electron and an ion. The resulting free electron is driven toward the electrode, generating electron avalanches along the way (Figure 9, steps 1 and 2). (2) Positive ions formed during the collisions drift toward the Faraday cup, whereas negative ions remain close to the anode surface (Figure 9, step 3). (3) Positive ion drift gives rise to a continuous unipolar current (corona onset current) measured by the nanocoulombmeter. (4) The positive ions remain on the Faraday cup, and the total charge accumulated is recorded as *Q*_residual_. (5) At higher humidities (increasing from 50 to 90%), larger water clusters are formed. These clusters have lower ionization potentials compared to common air constituents, which reduces the work required to generate ions. (6) Holding the voltage constant, the total positive space charge and ionizing collisions increase, and a greater number of positive ions contribute to the onset current at high humidity. (7) Ions remain in the Faraday cup after the electrode is removed (Figure 9, step 4).

A shortcoming of this proposed mechanism, however, is that it does not explain how metal–metal junctions contribute to the observed charge accumulation. We emphasize that the alligator clip by itself did not induce any charge accumulation, even though it also has sharp corners and teeth that should induce a strongly nonuniform electric field. It remains unclear what the mechanistic role of the metal–metal junction is in triggering or modulating charge accumulation at high humidity. We searched for correlations with different metal material properties (including conductivity, hardness, and work function) but did not find any meaningful correlation with the observed charging rates. Instead, a possible explanation, not directly tested here, is that minor variations in the electrode geometry play an outsized role in governing the initiation and magnitude of the corona onset current. Since the corona discharge begins at the electrode corners, where the necessary field strength for ionization is first met, the electrode geometry may be an important factor in charge accumulation. Although we took care to prepare the metal electrodes as similarly as possible, it is possible that the electrodes of different metals actually had slightly different curvatures at their corners and/or edges and yielded accordingly different currents. Additional experiments that systematically probe the impact of the electrode shape, including, for example, disc and needle geometries, are necessary to test this hypothesis.

Although the precise mechanism is not fully elucidated, there are clear practical implications for systems that use high voltage to manipulate lab-on-a-chip systems. For example, droplet electrophoresis at high voltages could be affected by the corona onset current if the laboratory humidity is sufficiently high. A scaling analysis provides an estimate whether this “extra charge” in the system might affect droplet charge acquisition experiments. Specifically, a standard droplet apparatus used in previous studies^9−11,16,18,19,23^ includes two parallel-plate electrodes, separated by a dielectric fluid, that are placed in a cuvette. The surface charge density on the positive high voltage electrode in the cuvette apparatus can be estimated using Gauss’ Law as σ = εε_o_*E*, where ε is the dielectric constant of the insulating oil between the electrodes, ε_o_ is the vacuum permittivity of space, and *E* is the electric field strength. For *E* ∼ 10^5^ V/m, ε ∼ 1, and ε_o_ ∼ 10^–11^ F/m, the induced surface charge density on the + HV electrode is estimated as σ_electrode_ ∼ 10^–6^ C/m^2^. Given that the positive ions generated by the corona onset current remain on the Faraday cup (cathode), we hypothesize that in the absence of a Faraday cup positive ions will remain on the grounded electrode side of the cuvette apparatus. Thus, the corona-induced surface charge density can be estimated by σ = *Q*/*A*, where *Q* is the total charge of positive ions from the corona onset current discharge and *A* is the surface area of the cuvette side. For *Q* ∼ 10^–9^ C, chosen to reflect the typical values observed in Figures 2–8, and a typical cuvette area, *A* ∼ 10^–4^ m^2^, the surface charge density on the cuvette is estimated as σ_cuvette_ ∼ 10^–5^ C/m^2^, a full order of magnitude larger than the charge directly induced on the electrode via application of the electric field. This conservative scaling estimate suggests that the generation of positive ions from the corona onset is not negligible, warranting further investigation of this possible confounding factor in droplet electrophoresis experiments.

There are several other complications. The above scaling analysis neglects the observed time dependence of the total positive charge generated during corona initiation; i.e., the accumulated residual charge in the cup increases with time. Additionally, this analysis assumes that positive ions generated by corona discharge are fixed on one side of the cuvette apparatus. Although the exact location of these ions is currently unknown, positive residual charges left near the cuvette apparatus may lead to deviations between the applied and measured electric field. As these charges accumulate, the time-dependent changes of the electric field imposed on the droplet will impact the acquired charge of the droplet and cause deviations from Maxwell’s theory. Future investigations are needed to quantify the effect of corona onset current on the electric field distribution of droplet charge acquisition apparatuses and the subsequent effect on a droplet’s acquired charge. Nevertheless, the observations discussed in this work offer a possible explanation to the reported change in the droplet acquired charge over time.^18,23^

## Conclusions

We have examined the charge of a metal alligator clip and metal electrode isolated in a Faraday cup at different applied potentials and relative humidity for 850 total trials. As the relative humidity increased, charge accumulation occurred and residual charge was left in the Faraday cup even after deactivation of the applied high voltage and physical removal of the electrode. This phenomenon was not specific to copper and stainless steel, as charge accumulation was observed between a copper alligator clip and copper, nickel, zinc, aluminum, and titanium electrodes. We rationalize our results in the context of corona onset discharge (dark discharge) and the subsequent unipolar steady current generated by collision-induced positive ion formation and drift toward the Faraday cup. The increase of charge rate with relative humidity was reported for all trials, and our findings agree well with the literature.

Although the detailed charging mechanism remains unclear, the results presented here lead to an important practical conclusion: ambient humidity can affect laboratory experiments in situations where one might not expect. To reduce undesirable variation in charge effects for high voltage systems using metal electrodes, e.g., microfluidic and lab-on-a-chip devices,^3−6,57^ researchers should conduct experiments under conditions of low ambient humidity. Neglecting the effects of ambient humidity could lead to unanticipated or erratic electrophoretic behavior. For practical applications, the results presented here are of fundamental interest for electrostatic precipitators and unipolar aerosol charging where relative humidity is known to affect gas discharge phenomena and electrostatic characteristics of devices.^39,54^
